# Prognostic Value of a Seven-Factor Risk Stratification System in T3N0 Colon Cancer: A Single-Center Retrospective Study

**DOI:** 10.3390/medicina62101846

**Published:** 2026-09-24

**Authors:** Abdussamet Çelebi, Eren Meral, Fırat Akagündüz, Ahmet Demirel, Mustafa Alperen Tunç, Burak Paçacı, Erkam Kocaaslan, Ali Kaan Güren, Selver Işık, Ezgi Çoban, Nazım Can Demircan, Osman Köstek, İbrahim Vedat Bayoğlu, Murat Sarı

**Affiliations:** 1 Department of Medical Oncology, Faculty of Medicine, Marmara University, Istanbul 34899, Türkiye; fratakagunduz0@gmail.com (F.A.); drahmetdemirel23@gmail.com (A.D.); m.alperen.tunc@gmail.com (M.A.T.); drpacaci@gmail.com (B.P.); erkamkocaaslan@gmail.com (E.K.); alikaanguren@gmail.com (A.K.G.); dr-selver83@hotmail.com (S.I.); ezgi.yuzugullu@gmail.com (E.Ç.); ncdemircan@gmail.com (N.C.D.); osmankostek@yahoo.com (O.K.); dr.vebay@gmail.com (İ.V.B.); 2 Department of Internal Medicine, Faculty of Medicine, Marmara University, Istanbul 34899, Türkiye; drerenmeral@gmail.com

**Keywords:** T3N0 colon cancer, stage IIA, risk scoring, tumor budding, intestinal obstruction, adjuvant chemotherapy, recurrence-free survival, disease-free survival, competing risks

## Abstract

*Background and Objectives*: This study aimed to validate the prognostic value of clinicopathologic risk factors and assess the discriminatory performance of a guideline-based seven-factor risk stratification system in T3N0 colon cancer. Secondary objectives included examining the independent prognostic role of mucinous histology and analyzing current adjuvant chemotherapy decision-making patterns. *Materials and Methods*: A total of 174 patients who underwent curative resection between 2015 and 2025 and were diagnosed with pathologic T3N0M0 (stage IIA) colon adenocarcinoma were retrospectively reviewed. Patients with T4 disease, rectal tumors, synchronous malignancies, neoadjuvant therapy and mismatch repair deficiency (dMMR/MSI-H) were excluded. Seven high-risk factors were defined: lymphovascular invasion (LVI), perineural invasion (PNI), intestinal obstruction, perforation, inadequate lymph node sampling (<12 lymph nodes), poor differentiation (Grade 3 and 4), and high-grade tumor budding (BD3, ≥10 buds according to ITBCC criteria). Mucinous histology was evaluated as a separate subgroup analysis. The primary endpoint was recurrence-free survival (RFS); secondary endpoints were disease-free survival (DFS) and overall survival (OS). Competing risks analysis was performed using the Aalen–Johansen estimator, with death without recurrence as the competing event. *Results*: The median follow-up was 43.7 months. Five-year RFS rates were 97.1%, 92.8%, and 70.0% in the low-, intermediate-, and high-risk groups, respectively (log-rank *p* = 0.041). Five-year OS rates were 87.2%, 88.6%, and 69.9%, respectively (log-rank *p* = 0.0004). Five-year cumulative incidence of recurrence in competing risks analysis was 2.9%, 7.1%, and 26.0%, respectively. Five-year DFS rates were 87.2%, 83.1%, and 54.2% (log-rank *p* = 0.0003). In multivariate DFS analysis, intestinal obstruction (HR = 4.26; 95% CI: 2.05–8.87; *p* < 0.001) and tumor budding (HR = 3.77; 95% CI: 1.67–8.53; *p* = 0.001) were independent prognostic factors; consistent but exploratory RFS estimates were observed (obstruction HR = 8.51; 95% CI: 2.50–28.96; *p* = 0.001; tumor budding HR = 5.48; 95% CI: 1.72–17.44; *p* = 0.004), derived from 12 recurrence events and therefore prone to overfitting. As a continuous variable, each 1-unit increase in the seven-factor risk score increased the RFS hazard 1.74-fold (95% CI: 1.21–2.51; *p* = 0.003; RFS C-index = 0.656; 95% CI: 0.489–0.830). A parsimonious two-factor model (obstruction + tumor budding) achieved a higher C-index of 0.760 (95% CI: 0.569–0.921) for RFS. Mucinous histology was present in 17 patients (9.8%) and showed no recurrence events. Adjuvant CT rates increased significantly with higher risk scores (*p* = 0.002). *Conclusions*: Guideline-based seven-factor risk stratification demonstrates statistically significant, though modest, discriminatory performance (RFS C-index = 0.656; 95% CI: 0.489–0.830; DFS C-index = 0.646; 95% CI: 0.519–0.778) in pMMR T3N0 colon cancer. Intestinal obstruction and tumor budding are independent prognostic factors that effectively stratify patients by recurrence risk; a parsimonious two-factor model based on these two features matched or exceeded the discrimination of the full score. The seven-factor system should be regarded as a validation of guideline-defined high-risk features that may support adjuvant chemotherapy discussions in stage IIA colon cancer; external validation is required before clinical implementation.

## 1. Introduction

Colorectal cancer is the third most commonly diagnosed cancer worldwide and the second leading cause of cancer-related death [1]. Stage II colon cancer (pT3–4N0M0) accounts for approximately 25–30% of all colorectal cancers, and the majority of these patients are diagnosed at the T3N0M0 stage [2,3]. Unlike patients with T4 tumors, those with T3N0 disease have relatively high 5-year overall survival rates after curative resection (approximately 89%) [4]; nevertheless, the role of adjuvant chemotherapy (CT) in this homogeneous patient group has been debated for decades [5,6].

Whereas the indication for adjuvant CT in T4 disease is clearly supported by guidelines, treatment decisions in T3N0 patients require a personalized approach based on clinicopathologic risk factors [7,8]. ASCO, NCCN, and ESMO guidelines have defined lymphovascular invasion (LVI), perineural invasion (PNI), intestinal obstruction, perforation, inadequate lymph node sampling (<12 lymph nodes), poor differentiation, and tumor budding (TB) as high-risk features in T3N0 disease [5,7,8]. However, the cumulative prognostic value of these factors in T3N0 patients and their individual contributions to adjuvant CT decision-making have not been adequately investigated [9,10].

Tumor budding (TB) has been standardized in recent years by international consensus guidelines (ITBCC 2016), and high-grade budding (BD3) has been shown to be an independent adverse prognostic factor in colorectal cancer [11,12]. This standardization has provided a foundation for incorporating TB into routine pathologic assessment and risk stratification. In contrast, the prognostic importance of mucinous histology remains controversial; although it is associated with poor prognosis in advanced-stage disease (stage III–IV), its independent prognostic role in stage IIA (T3N0) disease remains unclear [13,14].

Comprehensive studies evaluating the cumulative effect of multiple risk factors in T3N0 colon cancer and examining the distinct role of mucinous histology are limited. To address this gap, a retrospective cohort of patients with T3N0 colon cancer who underwent curative resection at our institution was assembled, and a guideline-based seven-factor risk stratification system derived from established ASCO/NCCN/ESMO criteria was evaluated. Mucinous histology was not included in the primary risk score; its independent prognostic role was examined through separate univariate analysis. The aims of this study were to evaluate the prognostic value of a scoring system based on seven clinicopathologic risk factors in T3N0 colon cancer, to examine the independent prognostic role of mucinous histology, and to analyze the adjuvant CT decision-making process in current clinical practice.

## 2. Materials and Methods

### 2.1. Patient Selection and Study Design

This study was conducted as a single-center retrospective cohort. Patients who underwent curative-intent surgical resection at our institution between 2015 and 2025 were retrospectively screened. Inclusion criteria were: (i) histopathologically confirmed primary colon adenocarcinoma; (ii) pathologic T3N0M0 (stage IIA, AJCC 8th edition) [15]; (iii) R0 curative resection; and (iv) adequate clinical and pathologic data. Patients with T4 disease, rectal tumors (including rectosigmoid), synchronous metastatic disease (M1), neoadjuvant therapy, non-curative resection (R1/R2), a history of other synchronous or metachronous primary malignancy, missing essential pathologic data and mismatch repair deficiency (dMMR/MSI-H status, determined by immunohistochemistry for mismatch repair proteins MLH1, MSH2, MSH6, and PMS2) were excluded. A total of 174 T3N0 patients were included in the study. Demographic data (age, sex), clinical features, tumor characteristics (location, T stage, grade, histologic type, LVI, PNI, lymph node count, tumor budding, MMR status), laboratory parameters, adjuvant CT receipt, and follow-up data (recurrence, mortality, follow-up duration) were recorded on a standardized data form. Somatic molecular testing (KRAS, BRAF, and EGFR) was not part of the routine clinical work-up of stage II colon cancer at our center during the study period; consequently, no patient in this cohort has documented mutation status, and the cohort is unselected with respect to these markers. Tumor location was grouped as right colon (cecum, ascending colon, hepatic flexure, transverse colon) and left colon (splenic flexure, descending colon, sigmoid).

### 2.2. Risk Stratification (Guideline-Based)

Seven high-risk factors recommended by current guidelines were evaluated: (1) lymphovascular invasion (LVI) positivity; (2) perineural invasion (PNI) positivity; (3) presentation with intestinal obstruction; (4) perforation; (5) inadequate lymph node sampling (<12 lymph nodes); (6) poor differentiation (Grade 3 and 4 [poorly differentiated]); and (7) high-grade tumor budding (BD3) according to ITBCC criteria [11,12]. Each factor present contributed 1 point, yielding a total risk score of 0–7. Tumor budding data were missing in 64 patients (36.8%) due to inconsistent pathologic reporting during the early study period; missing values were not imputed, and patients with missing tumor budding data were conservatively assigned a score of 0 for this factor (i.e., treated as budding-negative) in the risk scoring system. The subset with available tumor budding data comprised 110 patients (63.2%); in one of these patients, tumor grade was unknown and was conservatively scored as negative in the risk score, so that strictly complete data for all seven risk factors were available in 109 patients (62.6%). T4 disease was not included in the risk score because no patient met this criterion by study design. Patients were stratified into three risk categories according to the total score: low risk (score 0), intermediate risk (score 1–2), and high risk (score ≥ 3). Mucinous histology (adenocarcinoma containing >50% extracellular mucin) was not included in the primary risk score; instead, it was evaluated as an independent subgroup analysis. This decision was consistent with current guidelines, which do not list mucinous histology among the established high-risk features for adjuvant chemotherapy decisions in stage II colon cancer.

### 2.3. Adjuvant Chemotherapy

Adjuvant chemotherapy decisions were made considering risk factors, patient preferences, performance status, comorbidity, and physician assessment. Treatment assignment was not randomized in this retrospective study. Because no randomized trial has demonstrated the superiority of any specific adjuvant regimen—including oxaliplatin-containing combinations over fluoropyrimidine monotherapy—in stage II colon cancer, adjuvant CT receipt was analyzed as a binary variable (yes/no) rather than by regimen [5,16]. Individual regimens and the number of administered cycles were not systematically recorded in the study database; therefore, whether all patients received the same treatment protocol cannot be retrospectively confirmed, and regimen-specific or dose-intensity analyses were not performed.

### 2.4. Endpoints and Follow-Up

The primary endpoint was recurrence-free survival (RFS), defined as the time from surgery to confirmed local or distant recurrence; patients dying without confirmed recurrence were censored at the date of death [17,18]. Disease-free survival (DFS), defined as the time from surgery to recurrence, metastasis, or death from any cause, was evaluated as a secondary endpoint. Overall survival (OS), defined as the time from surgery to death from any cause, was evaluated as an additional secondary endpoint; patients alive at last follow-up were censored at the date of last contact. Recurrence was defined as clinically, radiologically, and/or pathologically confirmed local recurrence or distant metastasis.

### 2.5. Statistical Analysis

Statistical analyses were performed using SPSS software (version 25.0; IBM Corp., Armonk, NY, USA) and Python (version 3.11.6; Python Software Foundation, Beaverton, OR, USA). Continuous variables were presented as mean ± standard deviation or median (interquartile range, IQR); categorical variables were presented as counts and percentages. Group comparisons used the chi-square or Fisher exact test (categorical) and the Mann–Whitney U or Kruskal–Wallis test (continuous, according to distribution). DFS was estimated by the Kaplan–Meier method; between-group differences were assessed with the log-rank test. Because 61.3% of DFS events (19/31) were deaths without prior recurrence (i.e., competing events for cancer recurrence), the cumulative incidence of recurrence was also estimated using the Aalen–Johansen estimator within a competing risks framework, with death without recurrence treated as the competing event [19], using the lifelines (version 0.30.3) and SciPy (version 1.14.1) libraries in Python. Univariate and multivariate Cox proportional hazards regression was applied for prognostic factors; results were reported as hazard ratios (HRs) with 95% confidence intervals (CIs). Because of the limited number of events (DFS events *n* = 31), multivariate Cox models were restricted to a maximum of 2–3 variables in accordance with the 10:1 rule. Model discrimination was assessed with Harrell’s C-index. Factors influencing the probability of receiving adjuvant CT were analyzed with univariate and multivariate logistic regression; results were presented as odds ratios (ORs) with 95% CIs. Tumor budding data were missing in 64 patients (36.8%) due to inconsistent pathologic reporting during the early study period; missing TB values were not imputed, and patients with missing TB were conservatively assigned a score of 0 for this factor in the risk scoring system. Variables with *p* < 0.10 in univariate logistic regression were entered into the multivariable logistic model; complete-case analysis was used, excluding the 64 patients with missing tumor budding data (*n* = 110). The association between risk score and CT receipt was also examined with Spearman correlation and chi-square trend analysis. A two-sided *p* < 0.05 was considered statistically significant.

## 3. Results

### 3.1. Patient Characteristics

A total of 174 patients with pathologic T3N0M0 disease were included. The median age was 63.5 years (IQR: 53.2–73.0), and 64.4% of patients were male (112/174). The median follow-up was 43.7 months (IQR: 25.3–59.5). Tumors were located in the right colon in 44.8% and in the left colon in 55.2% of patients. All patients had pathologic T3 disease. The median number of harvested lymph nodes was 18.0 (IQR: 14.0–26.0). LVI was present in 51.7%, PNI in 16.1%, mucinous histology in 9.8% (*n* = 17), and Grade 3 and 4 (poorly differentiated) histology in approximately 17.8%. Adjuvant CT was administered to 34.5% of patients. During follow-up, 31 DFS events (12 recurrences, 19 deaths without recurrence) were observed (Table 1).

When right and left colon were compared, anemia and mucinous histology were more frequent in the right colon, and the number of harvested lymph nodes was higher on the right side. Right-sided patients were relatively younger. Left-sided patients more frequently presented with obstruction. These findings are consistent with previously reported biologic and epidemiologic differences between right- and left-sided colon cancers [20].

### 3.2. Distribution of Risk Factors

The prevalence of the seven risk factors is summarized in Table 2. The most frequent risk factor was LVI positivity (51.7%; 90/174), followed by intestinal obstruction (23.6%; 41/174), poor differentiation (Grade 3–4; 17.8%; 31/174), PNI positivity (16.1%; 28/174), inadequate lymph node sampling (12.6%; 22/174), and perforation (3.4%; 6/174). T4 tumors were excluded by design and were not present in the cohort. Tumor budding (BD3) was positive in 43 of 110 patients with available data (39.1%); data were missing in 64 patients (36.8%) and were not imputed.

The observed range of the seven-factor risk score was 0–6. Patient distribution by risk category was: low risk (score 0) *n* = 34 (19.5%), intermediate risk (score 1–2) *n* = 108 (62.1%), and high risk (score ≥ 3) *n* = 32 (18.4%).

Mucinous histology was present in 17 patients (9.8%). No recurrence was observed in the mucinous group. In univariate Cox analysis, mucinous histology was not associated with DFS (HR not estimable; log-rank *p* = 0.09). Accordingly, no independent prognostic value could be demonstrated for mucinous histology in this small subgroup. Mucinous histology was therefore not included in the primary scoring system.

### 3.3. Adjuvant Chemotherapy Distribution and Decision Determinants

In the overall cohort, the adjuvant CT rate was 34.5% (60/174). When CT receipt was examined by risk category, a significant association was found between risk score and CT receipt (*p* = 0.002). CT was administered to 11.8% of the low-risk group, 36.1% of the intermediate-risk group, and 53.1% of the high-risk group (Table 3; Figure 1). A positive correlation was observed between risk score and CT receipt. Among patients with available tumor budding data, the CT rate was higher in budding-positive (48.8%; 21/43) than budding-negative patients (28.4%; 19/67). Approximately one-third of patients with obstruction received CT (14/41). Because treatment allocation was non-randomized and influenced by the same risk factors under evaluation, these associations reflect real-world decision patterns and do not establish a causal effect of the risk score or of any individual factor on treatment outcomes.

In univariate logistic regression, LVI (OR 4.60; 95% CI 2.30–9.21), PNI (OR 4.46; 95% CI 1.90–10.45), and tumor budding positivity (OR 2.41; 95% CI 1.08–5.37) were significantly associated with CT receipt, whereas age > 65 reduced the probability of receiving CT (OR 0.35; 95% CI 0.18–0.67). In the multivariate model, LVI (OR 4.12; 95% CI 1.62–10.48; *p* = 0.003) and PNI (OR 3.43; 95% CI 1.02–11.53; *p* = 0.046) remained independently associated with CT receipt, whereas tumor budding did not retain independent significance after adjustment (OR 1.75; 95% CI 0.71–4.32; *p* = 0.221) (Table 4). Notably, obstruction—the strongest prognostic factor—did not guide the CT decision to a comparable degree. This observation points to a discordance between the strongest prognostic marker and the criteria most frequently used for treatment decisions [10].

**Table 4:** Factors influencing the adjuvant chemotherapy decision.

Variable	Univariate OR (95% CI)	*p*	Multivariate OR (95% CI)	*p*
LVI (+)	4.60 (2.30–9.21)	<0.001	4.12 (1.62–10.48)	0.003
PNI (+)	4.46 (1.90–10.45)	<0.001	3.43 (1.02–11.53)	0.046
Age > 65	0.35 (0.18–0.67)	0.002	0.39 (0.16–0.98)	0.046
Tumor budding (+) vs. (−)	2.41 (1.08–5.37)	0.031	1.75 (0.71–4.32)	0.221
Risk score (7F, continuous)	1.79 (1.33–2.42)	<0.001	—	—
Intermediate vs. low	4.24 (1.39–12.92)	0.011	—	—
High vs. low	8.50 (2.43–29.76)	<0.001	—	—

Variables with *p* < 0.10 in univariate analysis (LVI, PNI, tumor budding, age > 65) were entered into the multivariate logistic regression model. Multivariate analysis included 110 patients with complete tumor budding data. Risk score and risk-category analyses were univariate only, as the score is a composite of the individual factors. OR: Odds ratio; CI: confidence interval.

### 3.4. Survival Outcomes by Risk Category

As the primary endpoint, recurrence-free survival (RFS) considered confirmed cancer recurrence as the event of interest, with the 19 deaths without prior recurrence censored at the date of death. During follow-up, 12 recurrences (6.9%) were observed. The median time to recurrence was 15.2 months, and most recurrences (9 of 12; 75%) occurred within the first 24 months after surgery. The obstruction rate was markedly higher among patients who recurred than among those who did not (66.7% vs. 20.4%). By risk category, recurrence was observed in 1 of 34 low-risk (2.9%), 6 of 108 intermediate-risk (5.6%), and 5 of 32 high-risk patients (15.6%). Five-year RFS rates were 97.1% (95% CI 80.9–99.6%) in the low-risk, 92.8% (95% CI 84.1–96.8%) in the intermediate-risk, and 70.0% (95% CI 27.6–90.6%) in the high-risk group (overall log-rank *p* = 0.041) (Table 5; Figure 2). Pairwise comparisons demonstrated significant differences between the high- and intermediate-risk groups and between the high- and low-risk groups, whereas RFS did not differ between the low- and intermediate-risk groups.

Because a substantial proportion of DFS events (19 of 31; 61.3%) were deaths without prior recurrence, the cumulative incidence of cancer recurrence was also estimated within a competing risks framework using the Aalen–Johansen estimator, with death without recurrence treated as the competing event (Figure 3). Five-year cumulative incidence of recurrence was 2.9% in the low-risk, 7.1% in the intermediate-risk, and 26.0% in the high-risk group. These results confirm that the risk stratification system identifies patients at substantially higher risk of cancer recurrence, and that the prognostic separation of the high-risk category reflects a substantially higher rate of true cancer recurrence. Although the recurrence signal was consistent across the RFS, DFS, OS and competing-risks analyses, it is driven by a small number of events—five recurrences in the high-risk group versus seven across the remaining 142 patients—and chiefly separates the high-risk category, whereas the low- and intermediate-risk groups were statistically indistinguishable. These findings therefore require confirmation in larger cohorts.

As a secondary endpoint, 31 DFS events (12 recurrences and 19 deaths without recurrence) were observed. Five-year DFS rates according to the seven-factor risk categories were 87.2% (95% CI 64.2–95.9%) for low risk, 83.1% (95% CI 69.7–90.9%) for intermediate risk, and 54.2% (95% CI 23.7–77.1%) for high risk (overall log-rank *p* = 0.0003) (Table 5; Figure 2). Pairwise comparisons showed significant differences between the low- and high-risk groups and between the intermediate- and high-risk groups, whereas no difference was observed between the low- and intermediate-risk groups. None of the 17 patients with mucinous histology developed recurrence; in this subgroup, DFS did not differ significantly from that of non-mucinous tumors (log-rank *p* = 0.09).

Overall survival (OS), an additional secondary endpoint, was analyzed as the time from surgery to death from any cause. Five-year OS rates were 87.2%, 88.6%, and 69.9% in the low-, intermediate-, and high-risk groups, respectively (log-rank *p* = 0.0004) (Table 5; Figure 2). The high-risk group demonstrated significantly worse OS compared with the intermediate-risk group. Because only four high-risk patients remained at risk at 60 months, the 5-year estimates are accompanied by wide confidence intervals (Table 5); the corresponding 3-year estimates, based on larger numbers at risk, are provided as more robust summary statistics.

### 3.5. Prognostic Factors—Univariate and Multivariate Cox Regression

In univariate Cox regression analysis of RFS (12 recurrence events), intestinal obstruction (HR = 6.57; 95% CI: 1.98–21.82; *p* = 0.002) and high-grade tumor budding (BD3) (HR = 3.93; 95% CI: 1.25–12.39; *p* = 0.019) were the only factors significantly associated with recurrence. In multivariate analysis (Model 1R: obstruction + tumor budding), both remained independent prognostic factors: obstruction HR = 8.51 (95% CI: 2.50–28.96; *p* = 0.001) and tumor budding HR = 5.48 (95% CI: 1.72–17.44; *p* = 0.004). Because this model was fitted on 12 recurrence events with two covariates (approximately six events per variable, below the 10:1 threshold applied in this study), these estimates should be regarded as exploratory and are prone to overfitting, as reflected in the wide confidence intervals; they are presented as supportive of the more stable multivariate DFS estimates rather than as the primary multivariable result. When the risk score was examined as a continuous variable, each 1-unit increase raised the recurrence hazard 1.74-fold (95% CI: 1.21–2.51; *p* = 0.003; C-index = 0.656). Given the small number of recurrence events (*n* = 12), the non-significant univariate results for the remaining factors should be interpreted as potentially underpowered rather than as evidence of absence of a prognostic effect.

In univariate Cox regression analysis of DFS (Table 6), age among demographic factors showed borderline significance (HR = 1.03; 95% CI: 1.00–1.06; *p* = 0.061). Sex and tumor location (right vs left) were not associated with DFS (*p* = 0.806 and *p* = 0.815, respectively). Because no DFS events were observed among the 17 patients with mucinous histology, the Cox HR was not estimable; in a log-rank comparison, DFS of mucinous tumors likewise did not differ significantly from that of non-mucinous tumors (*p* = 0.09).

In individual analyses of the seven risk factors, intestinal obstruction emerged as the strongest prognostic factor for DFS (HR = 3.92; 95% CI: 1.90–8.10; *p* < 0.001). High-grade tumor budding (BD3) similarly worsened DFS significantly (HR = 3.29; 95% CI: 1.46–7.40; *p* = 0.004) (Figure 4). Perforation showed borderline significance (HR = 3.82; 95% CI: 0.89–16.45; *p* = 0.072). LVI, PNI, inadequate lymph node sampling, and Grade 3 and 4 differentiation did not reach statistical significance in univariate analysis (*p* = 0.567, *p* = 0.865, *p* = 0.128, and *p* = 0.195, respectively). Given the limited number of DFS events (*n* = 31), these non-significant findings should be interpreted as potentially underpowered rather than as evidence of absence of a prognostic effect.

When the risk score was examined as a continuous variable, each 1-unit increase raised the DFS hazard by 1.53-fold (95% CI: 1.18–1.97; *p* = 0.001; C-index = 0.646). By risk category, the hazard ratio for high versus low risk—in a comparison restricted to these two categories (*n* = 66)—was 5.04 (95% CI: 1.55–16.41; *p* = 0.007).

In multivariate Cox regression models (Table 7), two models were constructed for age and risk factors. In Model 1, obstruction and tumor budding remained independent prognostic factors after adjustment for age (obstruction HR = 4.26; 95% CI: 2.05–8.87; *p* < 0.001; tumor budding HR = 3.77; 95% CI: 1.67–8.53; *p* = 0.001). In Model 2, the risk score retained independent prognostic value after adjustment for age (HR = 1.54; 95% CI: 1.18–2.00; *p* = 0.001), whereas age was not significantly associated with DFS in this model (HR = 1.03; 95% CI: 1.00–1.06; *p* = 0.065).

The effect of adjuvant chemotherapy on DFS was evaluated by risk category. However, because of the retrospective design, treatment-selection bias, confounding by indication, and possible immortal-time bias, these findings are hypothesis-generating rather than causal. Because the CT-treated group comprised higher-risk patients, paradoxical HR values may be observed in crude comparisons; this does not imply that CT is harmful and must be interpreted with caution.

In univariate OS analysis, intestinal obstruction (HR = 3.83; 95% CI: 1.67–8.78; *p* = 0.002), perforation (HR = 5.55; 95% CI: 1.25–24.74; *p* = 0.025), high-grade tumor budding (BD3) (HR = 3.25; 95% CI: 1.25–8.48; *p* = 0.016), and age (HR = 1.05 per year; 95% CI: 1.01–1.09; *p* = 0.007) were significantly associated with OS. In multivariate OS analysis (obstruction + tumor budding + age), all three remained independently significant: obstruction HR = 4.00 (95% CI: 1.73–9.25; *p* = 0.001); tumor budding HR = 3.52 (95% CI: 1.35–9.19; *p* = 0.010); age HR = 1.05 (95% CI: 1.01–1.08; *p* = 0.015).

C-index comparison and sensitivity analysis: The seven-factor model achieved a C-index of 0.646 (95% CI: 0.519–0.778) for DFS prediction. A parsimonious two-factor model incorporating only the two independently significant factors (obstruction + tumor budding) achieved a slightly higher C-index of 0.708 (95% CI: 0.592–0.826). In a likelihood-ratio test comparing the model containing all seven individual risk factors with the two-factor model, adding the five remaining factors did not significantly improve model fit (LR χ^2^ = 2.71; df = 5; *p* = 0.745), indicating that these factors contribute little independent prognostic information beyond obstruction and tumor budding. For RFS, C-index values were 0.656 (95% CI: 0.489–0.830) for the seven-factor model and 0.760 (95% CI: 0.569–0.921) for the two-factor model. In a sensitivity analysis restricted to the 110 patients with available tumor budding data, the seven-factor model C-index for DFS increased to 0.764, confirming that missing TB data (36.8% of the cohort) substantially attenuated the apparent model discrimination when all 174 patients were included. Exploratory subgroup analyses revealed no significant difference in DFS between patients aged younger than 45 years and those aged 45 or older (*p* = 0.873), between left-sided and right-sided tumors (*p* = 0.815), or between adjuvant CT recipients and non-recipients (*p* = 0.803). The non-significant CT effect likely reflects confounding by indication in this retrospective setting, with higher-risk patients more frequently receiving adjuvant treatment.

## 4. Discussion

In this single-center retrospective study, the prognostic value of a scoring system based on seven clinicopathologic risk factors and the adjuvant CT decision-making process were evaluated in pathologic T3N0M0 colon cancer. Our principal findings are as follows: (i) recurrence-free survival (RFS) worsens as the risk score increases, with 5-year RFS declining from 97.1% in the low-risk group to 70.0% in the high-risk group (log-rank *p* = 0.041) and a corresponding 5-year cumulative incidence of recurrence of 26.0% in competing-risks analysis; (ii) intestinal obstruction and tumor budding emerged as independent prognostic factors for disease-free survival (DFS; obstruction HR = 4.26; tumor budding HR = 3.77), with consistent but exploratory recurrence-free survival (RFS) estimates (multivariate RFS: obstruction HR = 8.51; 95% CI: 2.50–28.96; *p* = 0.001; tumor budding HR = 5.48; 95% CI: 1.72–17.44; *p* = 0.004); (iii) no DFS events occurred among the 17 patients with mucinous histology, precluding any conclusion regarding its prognostic value in this subgroup; and (iv) in current clinical practice, the adjuvant CT decision is particularly associated with LVI, PNI, and TB, whereas obstruction does not adequately guide the CT decision. Our study is a validation and quantification effort rather than development of a novel system. The seven factors evaluated are established high-risk features recommended by major guidelines (ASCO, NCCN, ESMO) [5,7,8]. Our contribution lies in: (i) quantifying their cumulative prognostic impact in a homogeneous T3N0 cohort, (ii) demonstrating the independent roles of obstruction and tumor budding for both recurrence and survival outcomes, and (iii) providing real-world evidence on how these factors inform adjuvant CT decision-making in clinical practice.

The indication for adjuvant CT in T3N0 colon cancer is of particular importance because this group constitutes the most homogeneous subset of stage II disease. Although ASCO, NCCN, and ESMO guidelines recommend discussing adjuvant CT in the presence of high-risk features [5,7,8], the T3N0-specific prognostic weight of these factors and their combined effect have not been sufficiently clarified [9]. The seven-factor set used in our study largely overlaps with these guidelines. The low overall recurrence rate in our cohort (6.9%) reflects the relatively favorable prognosis of stage IIA disease; notably, death without recurrence accounted for the majority of DFS events (19 of 31), underscoring the importance of analyzing recurrence separately, with death as a competing event. In contrast, the decline of 5-year RFS to 70.0% in the high-risk group—with a corresponding decline of 5-year DFS to 54.2%—demonstrates a clinically meaningful separation.

The persistence of obstruction as an independent prognostic factor in multivariate analysis is noteworthy and consistent with the literature [21,22]. Obstruction was the strongest predictor of recurrence in our cohort (exploratory multivariate RFS: HR = 8.51; 95% CI: 2.50–28.96; *p* = 0.001) and remained independently associated with DFS after adjustment for age and tumor budding (Model 1: HR = 4.26; 95% CI: 2.05–8.87; *p* < 0.001). In patients presenting under emergency or semi-emergency conditions, tumor biology, systemic inflammatory response, suboptimal staging, and differences in surgical technique may worsen prognosis [23]. An important observation is that the rate of adjuvant CT receipt in the presence of obstruction did not increase in proportion to its prognostic weight. This finding suggests that obstruction should be made more visible in adjuvant treatment discussions in clinical practice.

The prognostic role of tumor budding has strengthened over the past decade [11]. After ITBCC 2016 standardization, the association of high-grade TB with independent poor prognosis has been demonstrated in numerous cohorts and meta-analyses [11,12]. In our study, high-grade tumor budding (BD3) significantly impaired DFS in both univariate (HR = 3.29; 95% CI: 1.46–7.40; *p* = 0.004) and multivariate analyses (Model 1: obstruction HR = 4.26, tumor budding HR = 3.77; *p* = 0.001). Inclusion of TB in the seven-factor score contributed to model discrimination. This finding supports the clinical utility of incorporating TB into routine pathology reports and risk scoring. Nevertheless, missing TB data in 36.8% of the cohort (64/174 patients) is an important limitation; sensitivity analysis restricted to patients with available TB data (*n* = 110) yielded a C-index of 0.764 for the DFS model, confirming that missingness substantially attenuated the apparent model performance in the full cohort. Systematic reporting of TB according to ITBCC criteria (BD1/BD2/BD3) in all pathology reports is essential for future external validation.

A noteworthy observation is that a parsimonious two-factor model using only obstruction and tumor budding (C-index = 0.708 for DFS; 0.760 for RFS) slightly outperformed the full seven-factor model (C-index = 0.646 for DFS; 0.656 for RFS). A likelihood-ratio test comparing the two models confirmed that the five additional factors did not significantly improve model fit (χ^2^ = 2.71; df = 5; *p* = 0.745). Accordingly, the seven-factor system is best regarded as a validation and quantification of established guideline-recommended high-risk features in a homogeneous T3N0 cohort rather than as the recommended clinical tool; the simplified obstruction-plus-tumor-budding model may be the more practical candidate for future evaluation, but neither model is ready for clinical use before external validation. This suggests that the five remaining factors (LVI, PNI, perforation, inadequate LN, Grade 3–4) contribute minimal independent discriminatory information in this pMMR T3N0 cohort, at least with the observed number of events. Furthermore, equal weighting of all seven factors is a methodologic limitation; obstruction and tumor budding demonstrate substantially higher prognostic weights than the other factors, and a weighted scoring approach may offer better discrimination. These observations require replication in larger external cohorts before clinical adoption of a two-factor approach.

Mucinous histology is a controversial prognostic factor: although generally associated with adverse outcomes in advanced-stage disease (stage III–IV), its role in stage II is debated, and some studies have even identified mucinous histology as a high-risk feature in stage II colorectal cancer [13,14]. In our study, mucinous histology was not associated with DFS; because no DFS events were observed among the 17 mucinous patients, the HR was not estimable due to zero events (log-rank *p* = 0.09). This finding should be interpreted cautiously: the absence of events in a small subgroup (*n* = 17) constitutes absence of evidence, not evidence of absence of a prognostic effect. The limited sample size precludes any definitive conclusion regarding the prognostic role of mucinous histology in pMMR T3N0 colon cancer, and the finding should not be misinterpreted as confirmation that mucinous histology is benign in this setting. Similarly, LVI and PNI were not significantly associated with DFS in univariate Cox analysis (LVI *p* = 0.567; PNI *p* = 0.865); as with mucinous histology, these null findings may reflect limited statistical power rather than a true absence of prognostic effect.

Logistic regression results indicate that, at our center, the adjuvant CT decision is shaped predominantly by LVI, PNI, and tumor budding. The increase in CT rates with rising risk score shows that a guideline-based risk-adaptive approach is partially implemented. Our study has important limitations: (i) the retrospective design introduces risks of treatment-selection bias and confounding by indication, and CT-treated and untreated groups may differ at baseline; (ii) the low number of events (31 DFS events, including 12 recurrences) limits statistical power, widens confidence intervals in multivariate models, and renders non-significant associations difficult to interpret; (iii) the single-center design may reduce external validity; (iv) the risk score has not been validated in an external cohort; (v) equal weighting of all seven factors is a simplification, as obstruction and tumor budding showed substantially higher prognostic weights; and (vi) no molecular markers (KRAS, BRAF, EGFR mutational status) were available in our dataset, limiting integration with current molecular risk stratification frameworks.

Our strengths include the assembly of a homogeneous T3N0 cohort (T4 disease and rectal tumors were systematically excluded, as were dMMR/MSI-H tumors), comprehensive evaluation of the seven risk factors, separate subgroup analysis of mucinous histology, and quantitative assessment of model performance by C-index comparison complemented by a likelihood-ratio test. This approach provides a defensible framework for risk stratification of a homogeneous T3N0 cohort.

Integration of risk stratification with circulating tumor DNA (ctDNA) may further refine adjuvant treatment selection in T3N0 disease. ctDNA-based detection of minimal residual disease accurately identifies patients at high risk of recurrence in stage II colon cancer [24], and the DYNAMIC trial demonstrated that a ctDNA-guided approach can reduce adjuvant CT use while maintaining recurrence control [25]. Systematic inter-center standardization of TB reporting (BD1/BD2/BD3) is a prerequisite for external validation of any TB-based risk score.

## 5. Conclusions

In this single-center retrospective validation study, a guideline-based seven-factor risk stratification system demonstrates statistically significant, though modest, prognostic discriminatory performance (RFS C-index = 0.656; 95% CI: 0.489–0.830; DFS C-index = 0.646; 95% CI: 0.519–0.778) in pMMR T3N0 colon cancer. Five-year RFS rates were 97.1%, 92.8%, and 70.0% in the low-, intermediate-, and high-risk groups, respectively (log-rank *p* = 0.041); five-year OS rates were 87.2%, 88.6%, and 69.9% (log-rank *p* = 0.0004). Intestinal obstruction and high-grade tumor budding (BD3) emerged as the strongest independent prognostic factors in both DFS and RFS analyses (multivariate DFS: obstruction HR = 4.26; 95% CI: 2.05–8.87; *p* < 0.001; tumor budding HR = 3.77; 95% CI: 1.67–8.53; *p* = 0.001; supportive but exploratory RFS estimates: obstruction HR = 8.51; 95% CI: 2.50–28.96; *p* = 0.001; tumor budding HR = 5.48; 95% CI: 1.72–17.44; *p* = 0.004). In competing-risks analysis, the five-year cumulative incidence of recurrence rose from 2.9% and 7.1% in the low- and intermediate-risk groups to 26.0% in the high-risk group, confirming that the prognostic separation of the high-risk category reflects a true excess of cancer recurrence. A parsimonious two-factor model (obstruction + tumor budding) matched or exceeded the discriminatory performance of the full seven-factor model (C-index = 0.708 vs. 0.646 for DFS; 0.760 vs. 0.656 for RFS), suggesting that a simplified scoring approach may be clinically practical. Mucinous histology showed no recurrence events in this small subgroup (*n* = 17); this absence of evidence should not be interpreted as evidence of no prognostic effect. The adjuvant chemotherapy decision in current practice is predominantly driven by LVI, PNI, and tumor budding, whereas obstruction—the strongest prognostic factor—is underweighted in treatment decision-making. Because of the retrospective design, single-center setting, limited event number, and missing tumor budding data in 36.8% of patients, these findings are hypothesis-generating. Although the recurrence signal was consistent across the RFS, DFS, OS and competing-risks analyses, it was driven by a small number of events and chiefly separated the high-risk category, whereas the low- and intermediate-risk groups were statistically indistinguishable. External cohort validation, prospective registries, and integration with molecular markers are required before clinical implementation of modified risk scoring systems.

## Figures and Tables

**Figure 1:**
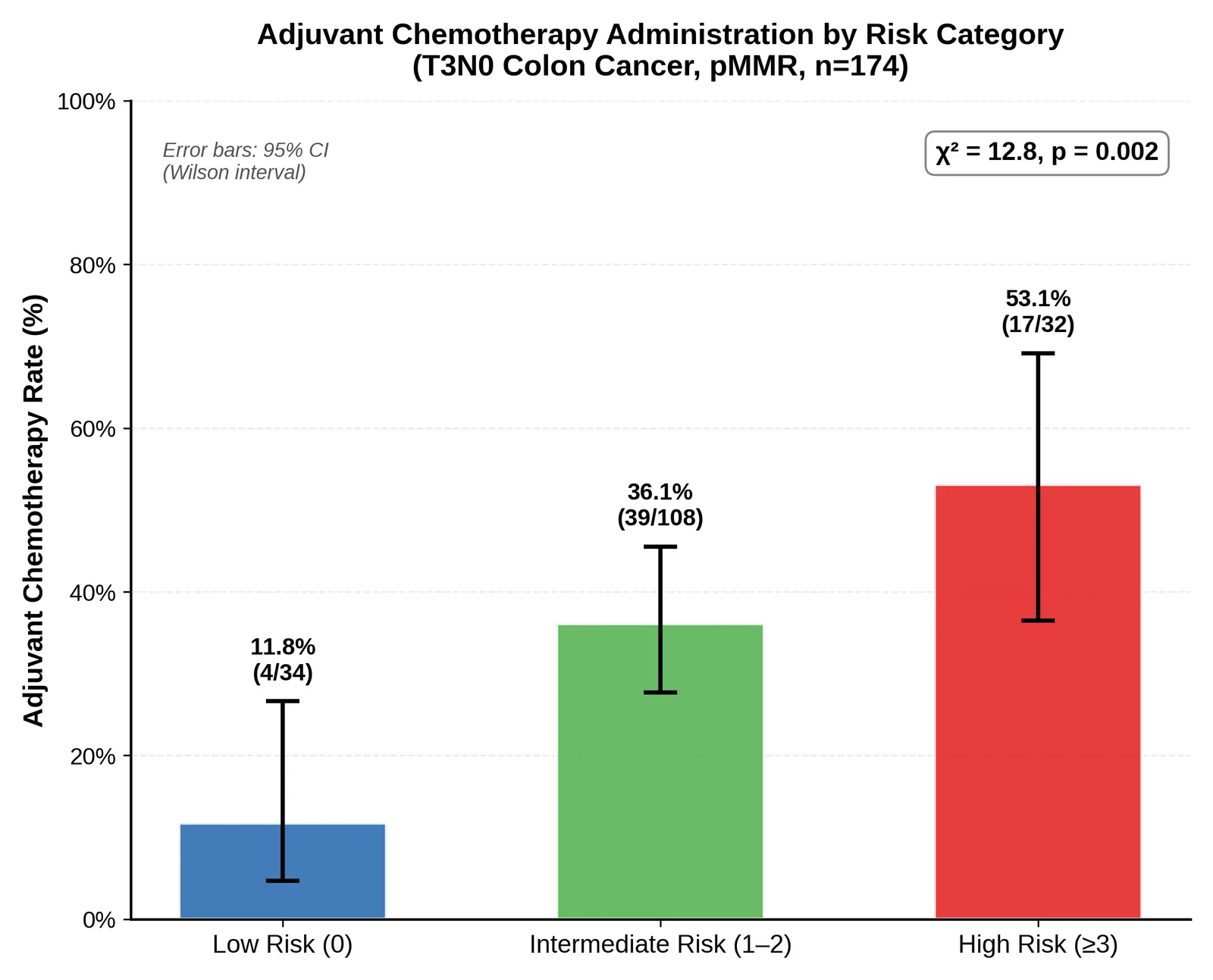
Adjuvant chemotherapy receipt by risk category (seven-factor risk score). Bars represent the proportion of patients receiving adjuvant CT in each risk group; error bars indicate 95% confidence intervals.

**Figure 2:**
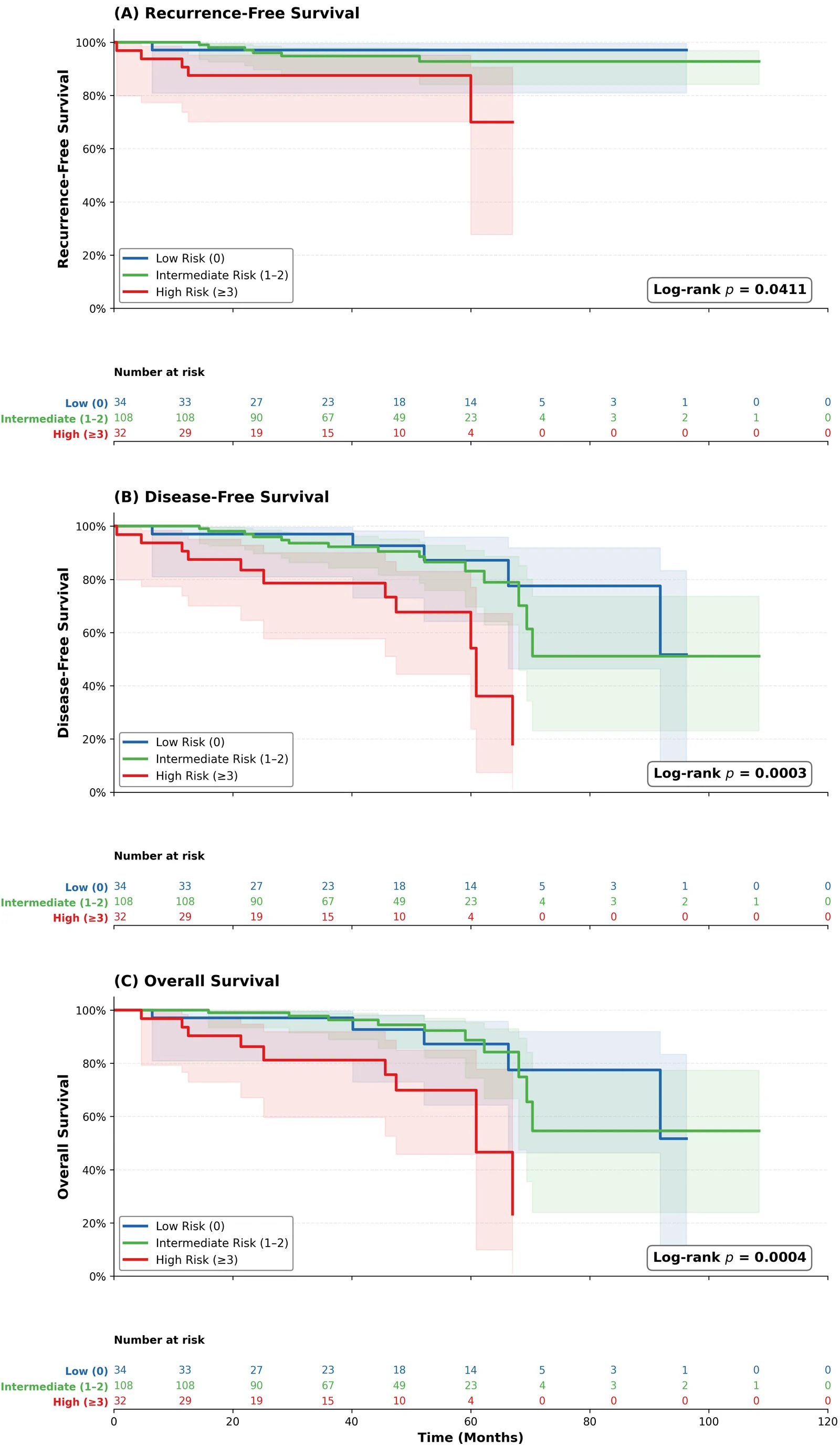
Survival outcomes by risk category (T3N0 colon cancer, pMMR, *n* = 174). Shaded areas represent 95% confidence intervals.

**Figure 3:**
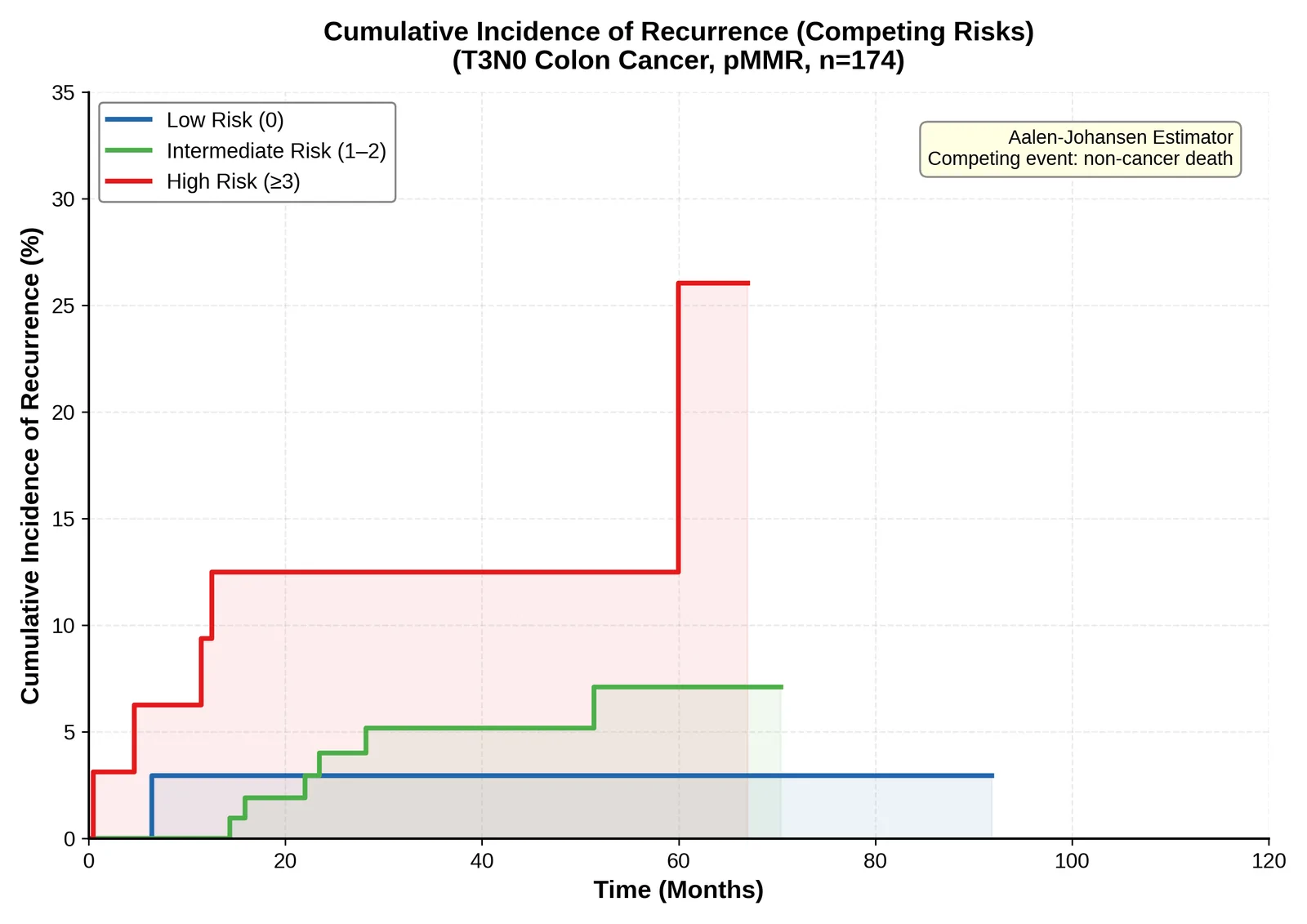
Competing risks analysis—cumulative incidence of cancer recurrence by risk category. Aalen–Johansen estimator; death without recurrence treated as competing event. Five-year cumulative incidence: 2.9% (low risk), 7.1% (intermediate risk), 26.0% (high risk).

**Figure 4:**
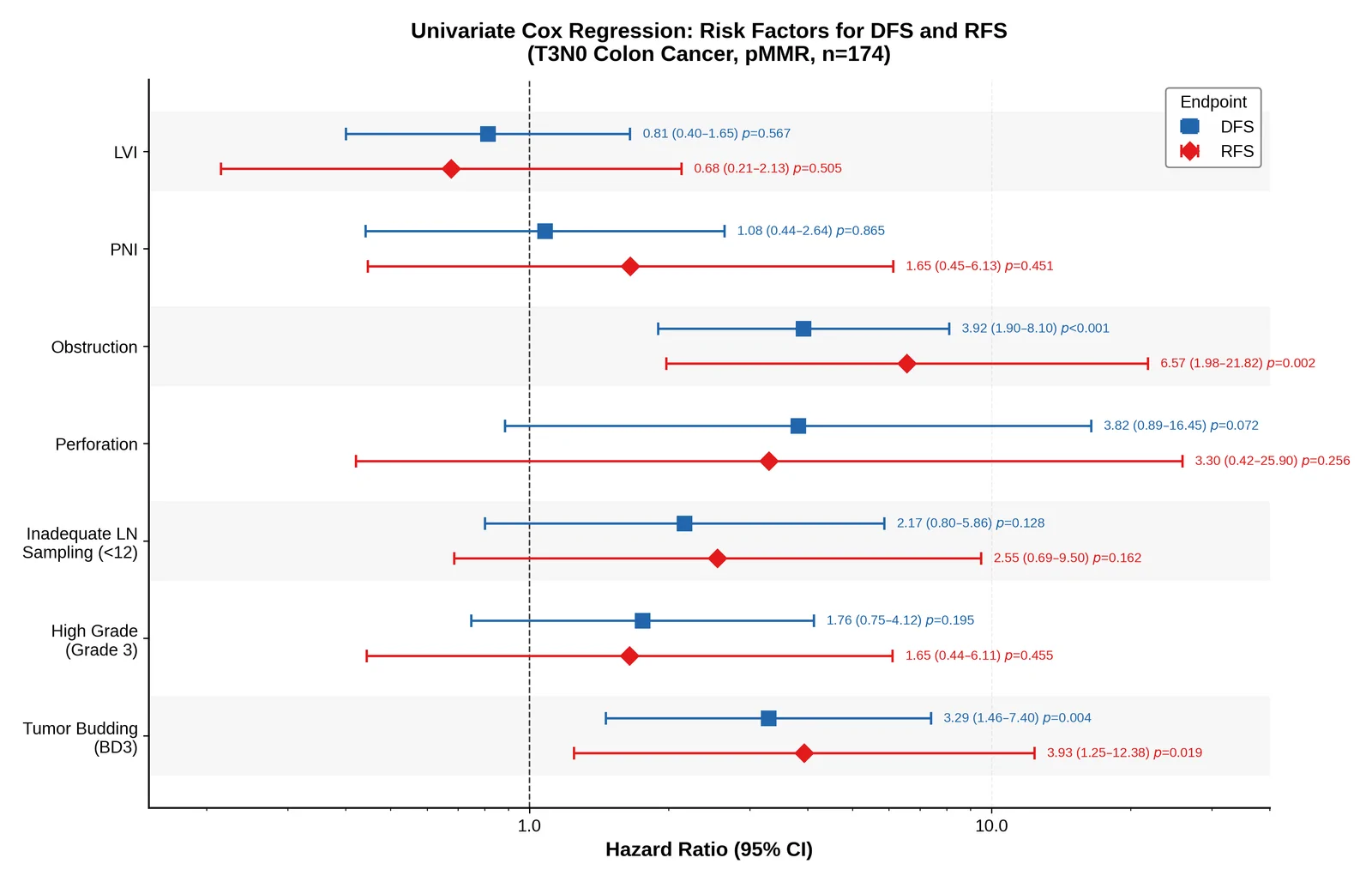
Univariate Cox regression analysis—forest plot (seven risk factors). The vertical dashed line indicates a hazard ratio of 1 (no effect).

**Table 1:** Patient characteristics (*n* = 174).

Characteristic	Value
Median age (IQR), years	63.5 (53.2–73.0)
Sex, male/female, *n* (%)	112 (64.4)/62 (35.6)
Median follow-up (IQR), months	43.7 (25.3–59.5)
Location, left/right, *n* (%)	96 (55.2)/78 (44.8)
Median harvested LN count (IQR)	18.0 (14.0–26.0)
LVI (+), *n* (%)	90 (51.7)
PNI (+), *n* (%)	28 (16.1)
Mucinous histology, *n* (%)	17 (9.8)
Grade 3 and 4 (poorly differentiated), *n* (%)	31 (17.8)
Obstruction, *n* (%)	41 (23.6)
Perforation, *n* (%)	6 (3.4)
Inadequate LN (<12), *n* (%)	22 (12.6)
High-grade tumor budding (BD3) *n* (%) *	43/110 (39.1)
Adjuvant CT, *n* (%)	60 (34.5)
Recurrence, *n* (%)	12 (6.9)
Mortality, *n* (%)	24 (13.8)

* Tumor budding was recorded from the pathology reports, in which positivity corresponded to high-grade (BD3) budding according to the reporting criteria used during the study period. Data were missing in 64 patients (36.8%) because the reports did not comment on tumor budding; missing values were not imputed, and the percentage was calculated among the 110 patients with known status. IQR: Interquartile range; LN: lymph node; LVI: lymphovascular invasion; PNI: perineural invasion; CT: chemotherapy.

**Table 2:** Distribution of the seven risk factors (*n* = 174).

Risk Factor (7F Scoring)	Present, *n* (%)	Absent, *n* (%)	Unknown, *n* (%)
LVI (+)	90 (51.7)	83 (47.7)	1 (0.6)
PNI (+)	28 (16.1)	145 (83.9)	1 (0.6)
Obstruction	41 (23.6)	132 (75.9)	1 (0.6)
Perforation	6 (3.4)	160 (92.0)	8 (4.7)
Inadequate LN (<12)	22 (12.6)	150 (86.2)	2 (1.1)
Grade 3 and 4	31 (17.8)	136 (78.2)	7 (4)
Tumor budding (BD3)	43 (24.7) *	67 (38.5)	64 (36.8)
Mucinous histology (subgroup—outside 7F)	17 (9.8)	156 (89.7)	1 (0.6)

* Percentages are based on the overall cohort (*n* = 174). Tumor budding was positive in 43 of 110 patients with known status (39.1%); data were missing in 64 patients (36.8%) and were not imputed. Mucinous histology was not included in the primary risk score and was evaluated as a separate subgroup analysis.

**Table 3:** Adjuvant chemotherapy distribution by risk category.

Risk Category	*n*	CT (+), *n* (%)	CT (−), *n* (%)	*p*
Low (0)	34	4 (11.8)	30 (88.2)	0.002 *
Intermediate (1–2)	108	39 (36.1)	69 (63.9)	
High (≥3)	32	17 (53.1)	15 (46.9)	
Total	174	60 (34.5)	114 (65.5)	

* Chi-square test across the three risk groups; *p* = 0.002.

**Table 5:** Survival outcomes by risk category (Kaplan–Meier estimates).

Endpoint	Risk Category	*n*	Events	Median Survival (Months)	1-Year (%)	3-Year (%)	5-Year (%)
**Recurrence-free survival** (primary)	Low (0)	34	1	Not reached	97.1	97.1	97.1
	Intermediate (1–2)	108	6	Not reached	100	95.8	92.8
	High (≥3)	32	5	Not reached	90.6	87.5	70
**Disease-free survival** (secondary)	Low (0)	34	5	Not reached	97.1	97.1	87.2
	Intermediate (1–2)	108	15	Not reached	100	93.6	83.1
	High (≥3)	32	11	60.9	90.6	78.6	54.2
**Overall survival** (secondary)	Low (0)	34	5	Not reached	97.1	97.1	87.2
	Intermediate (1–2)	108	10	Not reached	100	97.8	88.6
	High (≥3)	32	9	60.9	93.5	81.1	69.9

Events: RFS—confirmed cancer recurrence (deaths without recurrence censored); DFS—recurrence or death from any cause; OS—death from any cause. Overall log-rank test: RFS *p* = 0.041; DFS *p* = 0.0003; OS *p* = 0.0004. Pairwise comparisons for both RFS and DFS showed significant differences between the high-risk group and each of the other two groups (*p* < 0.05), with no difference between the low- and intermediate-risk groups. RFS: Recurrence-free survival; DFS: disease-free survival; OS: overall survival. Five-year Kaplan–Meier estimates with Greenwood 95% confidence intervals: RFS—low 97.1% (80.9–99.6%), intermediate 92.8% (84.1–96.8%), high 70.0% (27.6–90.6%); DFS—low 87.2% (64.2–95.9%), intermediate 83.1% (69.7–90.9%), high 54.2% (23.7–77.1%); OS—low 87.2% (64.2–95.9%), intermediate 88.6% (74.4–95.2%), high 69.9% (45.7–84.9%).

**Table 6:** Univariate Cox regression analysis for disease-free survival.

Variable	HR	95% CI	*p*
**Demographic and Clinical**			
Age (continuous)	1.03	1.00–1.06	0.061
Sex (Male vs. Female)	0.91	0.44–1.89	0.806
Location (Right vs. Left)	0.92	0.45–1.87	0.815
**Molecular Markers**			
Mucinous histology (+ vs. −)	—	—	0.09
**Risk Factors**			
LVI (+ vs. −)	0.81	0.40–1.65	0.567
PNI (+ vs. −)	1.08	0.44–2.64	0.865
Obstruction (+ vs. −)	3.92	1.90–8.10	<0.001
Perforation (+ vs. −)	3.82	0.89–16.45	0.072
Inadequate LN (<12) (+ vs. −)	2.17	0.80–5.86	0.128
Grade 3 and 4 (+ vs. −)	1.76	0.75–4.12	0.195
Tumor budding (BD3)	3.29	1.46–7.40	0.004
**Risk Scoring**			
Risk score 7F (continuous)	1.53	1.18–1.97	0.001
Intermediate vs. Low risk	1.39	0.49–3.91	0.533
High vs. Low risk	5.04	1.55–16.41	0.007

HR not estimable for mucinous histology (no DFS events among the 17 mucinous tumors); *p* value derived from the log-rank test. All hazard ratios are from univariate Cox proportional hazards regression. Intermediate vs. Low and High vs. Low risk comparisons were restricted to the respective two risk categories (*n* = 142 and *n* = 66). HR: Hazard ratio; CI: confidence interval.

**Table 7:** Multivariate Cox regression analysis for disease-free survival.

Model	Variable	HR	95% CI	*p*
Model 1	Obstruction	4.26	2.05–8.87	<0.001
	Tumor budding (BD3)	3.77	1.67–8.53	0.001
	Age (continuous)	1.02	0.99–1.05	0.118
Model 2	Risk score 7F (continuous)	1.54	1.18–2.00	0.001
	Age (continuous)	1.03	1.00–1.06	0.065

Model 1: Obstruction + TB (adjusted for age); Model 2: seven-factor risk score (adjusted for age); HR: hazard ratio; CI: confidence interval.

## Data Availability

The original contributions presented in this study are included in the article. Further inquiries can be directed to the corresponding author.

## Abbreviations

The following abbreviations are used in this manuscript:

AJCC	American Joint Committee on Cancer
ASCO	American Society of Clinical Oncology
BD3	Budding Grade 3
CI	Confidence Interval
C-index	Concordance Index
CT	Chemotherapy
ctDNA	Circulating Tumor DNA
DFS	Disease-Free Survival
dMMR	Deficient Mismatch Repair
ESMO	European Society for Medical Oncology
HR	Hazard Ratio
IQR	Interquartile Range
ITBCC	International Tumor Budding Consensus Conference
LN	Lymph Node
LR	Likelihood Ratio
LVI	Lymphovascular Invasion
MMR	Mismatch Repair
MSI-H	Microsatellite Instability-High
NCCN	National Comprehensive Cancer Network
OR	Odds Ratio
OS	Overall Survival
pMMR	Proficient Mismatch Repair
PNI	Perineural Invasion
RFS	Recurrence-Free Survival
TB	Tumor Budding
